# Antibodies Regulate Dual-Function Enzyme IYD to Induce Functional Synergy between Metabolism and Thermogenesis

**DOI:** 10.3390/ijms23147834

**Published:** 2022-07-15

**Authors:** Sunghyun Kang, Hwan-Woo Park, Kyung Ho Han

**Affiliations:** 1 Disease Target Structure Research Center, Korea Research Institute of Bioscience and Biotechnology (KRIBB), Daejeon 34141, Korea; skang@kribb.re.kr; 2 T-mac Co., Ltd., Daejeon 34141, Korea; 3 Department of Cell Biology, Konyang University College of Medicine, Daejeon 35365, Korea; hwanwoopark@konyang.ac.kr; 4 Myunggok Medical Research Institute, Konyang University College of Medicine, Daejeon 35365, Korea; 5 Department of Biological Sciences and Biotechnology, Hannam University, Daejeon 34054, Korea

**Keywords:** antibody, iodotyrosine deiodinase (IYD), dual function, antagonist, hypothyroidism

## Abstract

Iodotyrosine deiodinase (IYD) is a type of deiodinase enzyme that scavenges iodide from the thyroid gland. Previously, we showed that H3 Ab acts as an agonist on IYD to induce migration of cells to the heart and differentiate human stem cells into brown adipocyte-like cells. To continue this study, we investigated the dual function of IYD in hypothyroidism by blocking IYD and in thermogenesis by looking at the induction of brown adipocyte-like cells by treatment with H3 Ab in a mouse model. Surprisingly, our results suggest H3 Ab acts on IYD as both an antagonist and agonist to reduce T4 and increase core body temperature in the mouse model. Taken together, the data suggest IYD has a dual function that can regulate physiological metabolism and enhance thermogenesis.

## 1. Introduction

Each cell and protein operate by using a collection of different molecules that manage cooperative metabolism of the organism. To ensure the efficiency of the specific missions of a physiological task, some enzymes have dual functions to regulate metabolism, catalyze a substrate, or control a physiological reaction in the human body. Ceramide synthase is a dual function enzyme that catalyzes the production of vital lipids and turns lipid metabolism genes on or off [1]. Ketimine reductase has dual roles that degrade lysine in the brain and regulate thyroid hormone levels [2]. Spastin, a microtubule-severing enzyme, has a dual function of increasing the microtubule mass and expanding the microtubule network [3]. The dual function of CD73, ecto-nucleotidase, depends on a monoclonal antibody to inhibit immune suppression and induce an anti-tumor immune response [4].

Several functional agonist antibodies or affibody, which have been selected from combinatorial antibody libraries, are able to regulate cell differentiation fates [5,6,7,8]. The H3 antibody (H3 Ab) induces migration of cells to the heart and differentiates human stem cells into brown adipocyte-like cells, all while regulating the function of the enzyme, iodotyrosine deiodinase (IYD) [5]. IYD is known as a type of deiodinase enzyme that scavenges iodide by removing it from the thyroid gland. Iodide is used for the biosynthesis of thyroid hormone [9,10]. Via an enzyme reaction, iodine is configured to monoiodotyrosine (MIT) and diiodotyrosine (DIT) by binding to tyrosine residues. The coupling of two moieties of DIT makes thyroxine (T4). Linking one MIT with one DIT produces triiodothyronine (T3) [11,12,13,14]. Iodide homeostasis is crucial for the generation of thyroid hormone, which affects the function of all cells and organs.

Thyroid hormone plays a regulatory role in human body weight, internal temperature, and metabolism. Catalytic enzymes that control the thyroid hormone are located in the hypothalamus, white fat, brown adipose tissue (BAT), and skeletal muscle. Thyroid hormone exists in two main forms: thyroxine (T4) and triiodothyronine (T3). IYD plays an important role in thyroid hormone homeostasis by recycling iodine. T4 is the main form of thyroid hormone circulating in the blood. Lower than normal T4 levels suggest hypothyroidism, which can affect the heart and circulatory system in various ways [11,15,16,17].

Previously, we showed that H3 Ab acts as an agonist to IYD and induces differentiation of human stem cells into brown adipocyte-like cells and scavenges iodine from the thyroid hormone [5]. However, the antagonistic role of H3 Ab on the IYD enzyme has not yet been clarified. Our previous data suggest a possible role for H3 Ab in the biosynthesis of thyroid hormone since H3 Ab acts as an antagonist on the IYD enzyme and might contribute to the regulation of physiological metabolism.

Therefore, here, we focus on the physiological consequences of the enzyme acting as a classical enzyme and its role in thyroid function. These data suggest that when H3 Ab antagonizes the IYD enzyme, it, in turn, controls the generation of thyroid hormone and enhances thermogenesis.

## 2. Results

### 2.1. H3 Ab Induces Weight Gain

**Figure 1:**
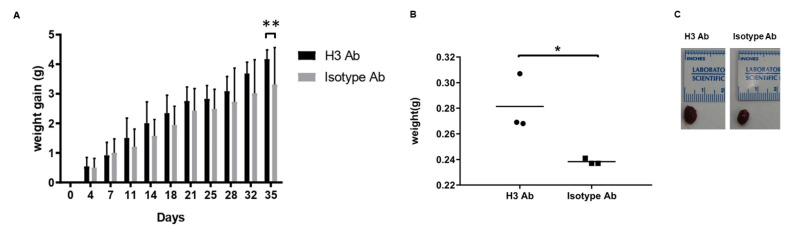

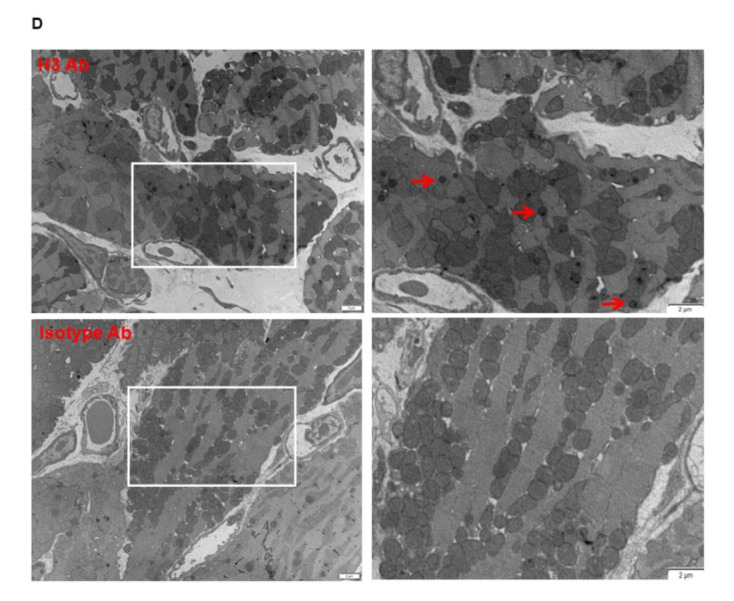
IYD inhibition by H3 Ab causes weight gain. (**A**) H3 Ab and isotype control antibody were injected (50 µg/mouse, *i.p.*, 2 times/week) into C57BL/6J mice for 3 weeks. Body weight was measured prior to treatment and then for 5 weeks. Significant differences between H3 Ab-treated and control mice on day 35 are indicated by ** *p* < 0.005 (Student’s *t*-test). Values are the mean ± s.d. for *n* = 7 mice. (**B**,**C**) H3 Ab and isotype control antibody were injected (50 µg/mouse, *i.p.*, 2 times/week) into C57BL/6J mice for 3 weeks. After euthanasia, mouse hearts were harvested and weighed and measured. Significant differences between H3 Ab-treated and control mice are indicated by * *p* < 0.05 (Student’s *t*-test). Values are the mean ± s.d. for *n* = 3 mice. (**D**) After H3 Ab (top) or isotype control antibody (bottom) were injected (50 µg/mouse, *i.p.*, 2 times/week) into C57BL6 mice for 3 weeks, the hearts were harvested and scanned by electron microscopy. The white squares on the pictures on the left indicate the areas that have been magnified on the corresponding images on the right. Red arrows indicate lipid droplets. Scale bars = 1 or 2 μm.

A genetic defect in IYD is known to be a cause of congenital hypothyroidism, a condition characterized by increased weight gain. Therefore, we were interested in determining whether treatment with H3 antibody would also yield increased weight gain. To this end, we injected H3 Ab or isotype control antibody (50 µg/mouse, *i.p.*, 2 times/week) into wild-type C57BL/6J mice for 3 weeks and measured body weight twice a week. We found that H3 Ab-treated mice gained significantly more weight compared to the control group (Figure 1A).

Since H3 Ab induced cell migration from the bone marrow to the heart [5], we also measured the heart size from sacrificed mice on day 35 of the body weight experiment. We showed that H3 Ab-treated mice gained significantly more body weight and heart weight compared to the control group (Figure 1B,C). In addition, we scanned the heart sections by electron microscopy to look at the lipid droplets in the mouse hearts and found that the mice treated with H3 Ab had more lipid droplets than the controls (Figure 1D).

H3 Ab has already been shown to inhibit the function of the enzyme, IYD, and these new data suggest this inhibition contributes to body weight gain characteristic of hypothyroidism.

### 2.2. H3 Ab Controls Thyroid Hormone

**Figure 2:**
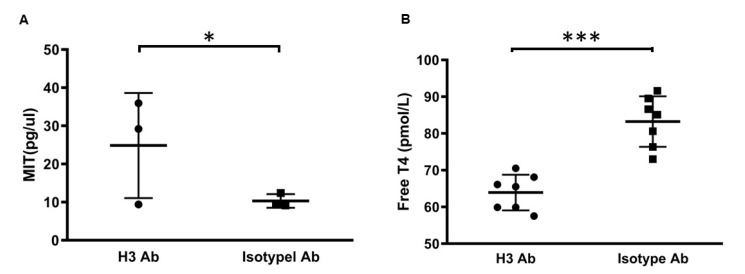
IYD blocking by H3 Ab. (**A**) H3 Ab or isotype control antibody was injected (50 µg/mouse *i.p.* 2 times/week) into C57BL/6J mice. After 3 weeks, urine samples from mice were analyzed by HPLC LC/MS. Significant differences between H3 Ab-treated and control mice are indicated by * *p* < 0.05 (Student’s *t*-test). Values are the mean ± s.d. for *n* = 3 mice. (**B**) H3 Ab or isotype control antibody was injected (50 µg/mouse, *i.p.*, 2 times/week) into C57BL/6J mice. After 3 weeks, blood serum was taken from mice and tested for free T4. Significant differences between H3 Ab-treated and control mice are indicated by *** *p* < 0.0005 (Student’s *t*-test). Values are the mean ± s.d. for *n* = 7 mice.

High levels of iodine in the body lead to increased ratios of DIT/MIT and T4/T3 whereas iodine deficiency has the opposite effect [18]. Since H3 Ab blocks the function of IYD, we hypothesized that iodine salvaging by IYD would be impaired, leading to increased levels of MIT substrate and decreased levels of T4 [13,14]. To this end, we investigated whether H3 Ab could be used to induce more mono-iodotyrosine (MIT), which is a substrate for IYD, in C57BL/6J mice. H3 Ab was injected (50 µg/mouse, *i.p.* two times/week) into C57BL/6J mice. After 3 weeks, urine was collected and analyzed by HPLC LC/MS. We showed that H3 Ab-treated mice had significantly increased MIT levels compared to mice from the control group (Figure 2A).

Low levels of free thyroxine (T4), resulting from less available iodine, are another feature of hypothyroidism, so we next studied the ability of H3 Ab treatment to reduce free T4 levels in the serum. H3 Ab was injected (50 µg/mouse, *i.p.*, 2 times/week) into C57BL/6J mice, and after 3 weeks, blood was collected and analyzed by radioimmunoassay. We showed that H3 Ab-treated mice had significantly decreased free T4 levels compared to mice from the control group (Figure 2B).

Collectively, these results suggest that H3 Ab blocks the function of IYD and plays a major role in hypothyroidism metabolism.

### 2.3. H3 Ab Increases Core Body Temperature

**Figure 3:**
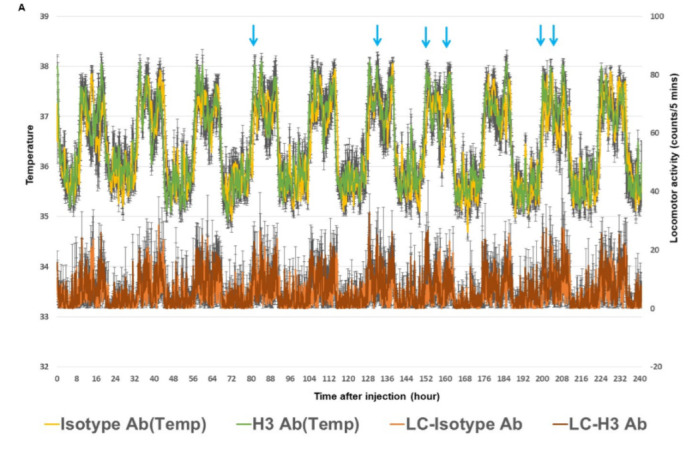

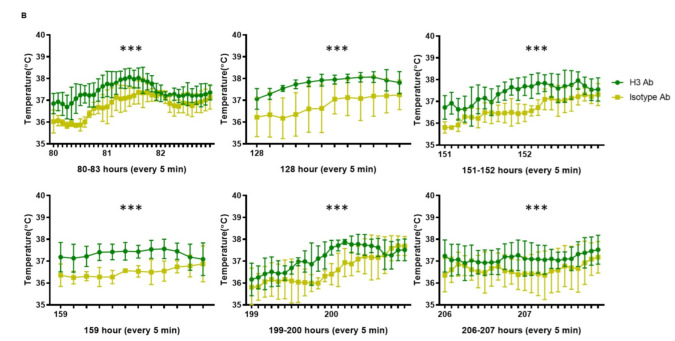
H3 Ab induces thermogenesis. Radiotelemetry devices were implanted into the mice. Following the surgical implantation and recovery period, H3 Ab or isotype control antibody was injected (50 µg/mouse, *i.p*., 2 times/week) into C57BL/6J mice for 3 weeks, and temperature data from mice were recorded by the automated data acquisition system. (**A**) The upper graph indicates body temperature recorded for the first 10 days after 3 weeks of treatment with antibody. The blue arrows indicate time points where mice experienced a significantly increased body temperature. The lower graph indicates locomotor activity (LC). (**B**) Raw temperature data from the statistically significant time points indicated by blue arrows in (**A**). Significant differences are indicated by *** *p* < 0.0005 (two-way ANOVA). Values are the mean ± s.d. for *n* = 5 mice.

In our previous study, we showed that H3 Ab could transform stem cells into brown adipocyte-like cells [5]. Brown adipose tissue contributes to thermogenesis and plays an important role in controlling body temperature. To extend these data, we next tested whether increased brown adipose tissue differentiation by H3 Ab would lead to an increase in core body temperature by measuring body temperature over time via radiotelemetry devices implanted into the mice. To do this, mice were injected with H3 Ab (50 µg/mouse, *i.p*., 2 times/week) for 3 weeks at 25 ± 0.5 °C. The group of animals treated with H3 Ab showed a significantly increased body temperature compared to control mice (Figure 3A,B). These results suggest that transformed brown adipocyte-like cells induced by treatment with H3 Ab contribute to an increased core body temperature and play an important role in thermogenesis.

### 2.4. The Selected H3 Antibody Has Dual Function

**Figure 4:**
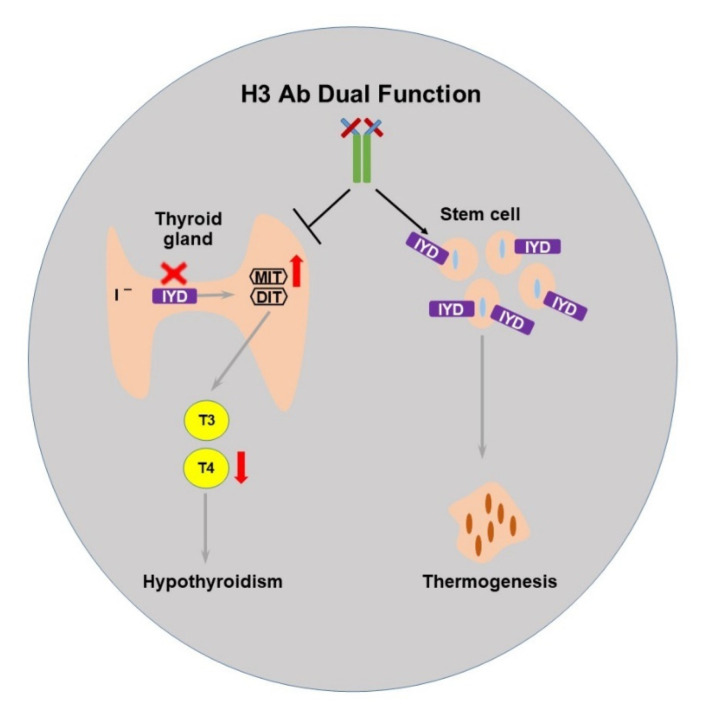
Proposed scheme for the dual function of IYD by H3 Ab. IYD scavenges iodine and, in the presence of the MIT/DIT substrate, catalyzes the production of triiodothyronine (T3) and thyroxine (T4) thyroid hormones. H3 Ab can block the IYD enzyme, which leads to increased MIT substrate, less iodine scavenged, and decreased T4 levels. IYD-expressing stem cells function as a receptor for H3 Ab, and binding of H3 Ab to IYD induces differentiation of the stem cells into brown adipocytes with increased lipid droplets.

Previously, we presented IYD as a metabolic enzyme in thyroid cells that works as a receptor for human stem cell differentiation. To further examine the function of H3 Ab on IYD, we performed in vivo studies by treating mice with H3 Ab or control antibody. The results showed significant reductions in T4 and weight gain in H3 Ab-treated mice compared to controls (Figure 1 and Figure 2). The treatment also significantly increased body temperature (Figure 3). These data suggest IYD has a dual function in the body and binding of H3 Ab to IYD in the thyroid gland and stem cells reveals the role of IYD in hypothyroidism and thermogenesis (Figure 4).

## 3. Discussion

Previously, we have shown that the iodotyrosine deiodinase (IYD) enzyme not only scavenges iodine from halogenated by-products of thyroid hormone production but also acts as a receptor on bone marrow stem cells to induce differentiation of brown adipocyte-like cells [5]. Thus, we suggested H3 Ab acts as an agonist to induce migration of cells to the heart and differentiate human stem cells into brown adipocyte-like cells. To continue this study, here, we investigated the dual function of IYD in hypothyroidism by blocking IYD and in thermogenesis by looking at the induction of brown adipocyte-like cells by H3 Ab treatment in a mouse model. Remarkably, our results suggest H3 Ab acts on IYD as both an antagonist and agonist molecule to regulate thyroid metabolism and body temperature.

In the previous and current studies, we report that agonist anti-IYD antibody can induce stem cells to differentiate into brown adipocyte-like cells and increase body temperature, whereas antagonist anti-IYD antibody reduces thyroid hormone. Thus, we presumably suggest IYD has a dual function to induce functional synergy between metabolism and thermogenesis. H3 Ab can block the IYD enzyme, which leads to increased MIT substrate, less iodine scavenged, and decreased T4 levels. IYD-expressing stem cells function as a receptor for H3 Ab, and binding of H3 Ab to IYD induces differentiation of the stem cells into brown adipocyte-like cells. Brown adipose tissue contributes to thermogenesis. Despite our finding that H3 Ab interacts with IYD to regulate physiological metabolism and enhance thermogenesis, we did not investigate the potential indirect interactions of H3Ab–IYD enzyme. Thus, further studies will help us to better understand the detailed mechanism. Thyroid hormone plays a crucial role in normal development and metabolic homeostasis during human life. In order to regulate thyroid hormone, catalytic enzymes are located in the hypothalamus, white fat, brown adipose tissue (BAT), and skeletal muscle. Thyroid hormone exists in two main forms: thyroxine (T4) and triiodothyronine (T3). T4 is the main circulating hormone in the blood. Low T4 levels indicate hypothyroidism, which can affect the heart and circulatory system in various ways [11,15,16,17,19,20]. Despite our finding that H3 Ab-treated mice had significantly decreased free thyroxine (T4) levels, resulting from less available iodine, another feature of hypothyroidism, we cannot rule out the involvement of off-target effects of H3 Ab that may have indirectly decreased iodine without blocking the function of IYD. Therefore, further studies, including in vivo experiments performed under low-iodine conditions, are needed to better understand the detailed mechanism of H3 Ab and how it regulates IYD, physiological metabolism, and thermogenesis.

Brown adipocytes have been reported to have the ability to dispel heat in energy homeostasis when in a cold environment. Additionally, brown adipose tissue plays a crucial role in non-shivering thermogenesis, which requires energy. These thermogenic adipocytes are considered to have unique developmental origins, bioenergetics, and physiological functions. Thermogenic adipocytes use lipid and glucose to regulate body temperature [21,22]. Consistent with previous studies, our results support that H3 Ab induces stem cells to differentiate into brown adipocyte-like cells, and these cells contribute to increased production of heat energy and core body temperature via non-shivering thermogenesis at room temperature.

These data suggest that human pluripotent stem cells have the potential to differentiate into brown adipocytes and be used to treat metabolic disease. Other groups have shown that in the presence of transplanted human brown adipocytes, recipient mice showed significantly higher metabolic activity and thermogenic activity and decreased blood glucose levels [23,24,25,26]. Interestingly, we did not observe any significant difference in the glucose levels between H3 Ab-treated mice and control mice (data not shown).

In summary, using H3 Ab, we uncovered an unexpected dual function of IYD in metabolism and thermogenesis. Although the mechanistic process by which this thermogenesis occurs remains undefined, our evidence suggests that IYD is an enzyme with dual function and that H3 Ab acts as an antagonist and agonist to reduce T4 and increase core body temperature in the mouse model.

## 4. Materials and Methods

### 4.1. Study Design

Our study was designed to investigate the dual function of IYD in hypothyroidism by blocking IYD and in thermogenesis by looking at the induction of brown adipocyte-like cells by H3 Ab in a mouse model. Several in vivo approaches were used to determine whether H3 Ab acts on IYD as both an antagonist and agonist to reduce T4 and increase core body temperature in the mouse model.

### 4.2. Mice and Cell Lines

C57BL/6J (The Jackson laboratory, Bar Harbor, ME, USA) mice were used for all experiments. Mice were housed and handled at 20 to 26 °C according to protocols approved by the Institutional Animal Care and Use Committee at The Scripps Research Institute and Hannam University (12-0029/HNU2021-6). The Expi293F cell line was maintained in Expi293 Expression Media (Gibco-Invitrogen, Waltham, MA, USA).

### 4.3. Telemetry

Mice were anesthetized with isoflurane (induction 3–5%) and surgically implanted with radiotelemetry devices (TA-F10, Data Sciences, St. Paul, MN, USA) into the peritoneal cavity to measure core body temperature (CBT) and activity. Following surgical implantation and appropriate wound closure, the animals were allowed to recover for 2 weeks prior to recording telemetry data from freely moving animals. Each mouse was individually housed in a cage in a room maintained at 25 ± 0.5 °C. The cages were positioned onto the receiver plates (RPC-1; Data Sciences, St. Paul, MN, USA) and radio signals from the implanted transmitter were recorded every 5 min with a fully automated data acquisition system (Dataquest ART, Data Sciences, St. Paul, MN, USA).

### 4.4. Treatment with Isolated Antibody

Mice were treated *i.p.* with H3 Ab or Isotype control antibody (Bioxcell, Lebanon, NH, USA) (50 µg/mouse) two times per week. Treatments were initiated at 6 weeks of age, and the experiment was terminated at 11 weeks of age.

### 4.5. MIT Assay

Urine samples were analyzed for MIT levels using HPLC LC/MS analysis, which was performed as described previously [13,27]. After H3 Ab were injected (50 µg/mouse, *i.p.*, 2 times/week) into C57BL6 mice for 3 weeks, to each 100 µL of mouse urine, 60 µL of internal standard solution was added. After vortexing, pooled urine samples were evaporated to dryness and reconstituted in 100 µL of 2% acetonitrile in water. A 10-µL aliquot of the final sample was injected for HPLC-LC/MS analysis. Chromatographic separation was achieved on an HPLC System (ThermoFinnigan LTQ Ion Trap, San Jose, CA, USA) interfaced to the mass spectrometer for automated LC-MS analyses. The sample was eluted at a flow rate of 300 µL/min and a linear gradient of 5 min: start = 97% eluant A (0.1% formic acid in water), 3% eluant B (acetonitrile/water: 9/1); end 100% eluant B. The system was held for 3 min with the initial eluant at a flow rate of 500 µL/min to equilibrate the column; the column temperature was maintained at 20 °C. The mass spectrometer had a nano-ion source with a 2 kV electrospray voltage (The Scripps Center for Mass Spectrometry, Cincinnati, OH, USA). The source temperature was set at 85 °C; the optimal cone voltage ranged from 30–40 V for the analytes and IS. MIT were measured by multiple reaction monitoring (MRM) using transitions *m/z* 364 → *m/z* 135 for MIT with an optimal collision energy of 42 eV. Settings were optimized using a 0.1-µmol/liter solution of MIT in water and urine.

### 4.6. Free T4 Assay

Free T4 assay dialysis was performed with 200 µL of serum. After dialysis, an aliquot of the dialysate was incubated in a specific antibody-coated tube with radiolabeled tracer for 3 h at 37 °C. At the end of the incubation period, the tube was washed to remove any unbound components, and the radioactivity bound to the solid phase was measured in a gamma counter.

### 4.7. Statistical Analysis

The data are expressed as the mean ± SD. Statistical significance was determined using an unpaired two-tailed Student’s *t*-test or two-way ANOVA followed by a post hoc test. *p* values of <0.05 were considered significant.
